# The effect of *RHIZOMA COPTIDIS* and *COPTIS CHINENSIS* aqueous extract on radiation-induced skin injury in a rat model

**DOI:** 10.1186/1472-6882-13-105

**Published:** 2013-05-15

**Authors:** Xi-Jing Wang, Shuai Lin, Hua-Feng Kang, Zhi-Jun Dai, Ming-Hua Bai, Xiu-Long Ma, Xiao-Bin Ma, Meng-jie Liu, Xiao-Xu Liu, Bao-Feng Wang

**Affiliations:** 1 Department of Oncology, the Second Affiliated Hospital of Xi’an Jiaotong University, Xi’an, 710004, China; 2 Department of Oncology, the First Affiliated Hospital of Xi’an Jiaotong University, Xi’an, 710061, China

**Keywords:** *RHIZOMA COPTIDIS*, *COPTIS CHINENSIS*, Radiation, Skin injury

## Abstract

**Background:**

Radiation-induced skin injury is a common complication of radiotherapy. The *RHIZOMA COPTIDIS* and *COPTIS CHINENSIS* aqueous extract (RCE) can ameliorate radiation-induced skin injury in our clinical observation. But, the protective mechanism of *RHIZOMA COPTIDIS* and *COPTIS CHINENSIS* in radiation-induced skin injury remains unclear.

**Methods:**

In this experiment, we developed a radiation-induced skin injury rat model to study the mechanism. The animals were randomly divided into control group, treatment group, radiation group, and treatment and radiation group. 5 rats in each group were separately executed on 2 d and 49 d post-radiation. The semi-quantitative skin injury score was used to measure skin reactions by unblinded observers, and hematoxylin and eosin staining was used to evaluate the damage areas by irradiation. The MDA content, SOD activity of skin and serum were measured to detect the oxidative stress.

**Results:**

Acute skin reactions were caused by a single dose of 45 Gy of β-ray irradiation, and the skin injury could be found in all rats receiving irradiation based on the observation of HE staining of skin at different time-points, while RCE could significantly ameliorate those changes. The MDA content in serum and skin of control rats was 4.13 ± 0.12 mmol/ml and 4.95 ± 0.35 mmol/mgprot on 2 d post-radiation. The rats receiving radiation showed an increased content of MDA (5.54 ± 0.21 mmol/ml and 7.10 ± 0.32 mmol/mgprot), while it was 4.57 ± 0.21 mmol/ml and 5.95 ± 0.24 mmol/mgprot after treated with RCE (p < 0.05). Similar changes of the MDA content could be seen on 49 d post-radiation. However, the SOD activity of rats receiving radiation decreased compared with control group on both time-points, which was inhibited by RCE (p < 0.05). Meanwhile, no valuable changes could be found between control group and treatment group on 2 d and 49 d.

**Conclusions:**

Our study provides evidences for the radioprotective role of RCE against radiation-induced skin damage in rats by modulating oxidative stress in skin, which may be a useful therapy for radiation-induced skin injury.

## Background

Radiotherapy is a useful modality for cancer therapy, but ionizing radiation may injure surrounding normal tissues [1,2]. The effect of ionization caused by radiation can kill the tumor cell directly or indirectly by generating reactive oxygen species (ROS), free radicals (FR) to cure the disease. The skin is primarily affected by the production of ROS and FR, release of inflammatory mediators/cytokines [3-5], and the skin may be significantly injured and its function profoundly impaired during radiation therapy [6,7]. Although we endeavor to maximize anti-cancer effects and minimize skin toxicity, radiation-induced skin injury is the most common complication of radiotherapy, with inflammatory damage caused by radiation on the skin, which may lead to treatment interruptions.

Under normal conditions, the deleterious effects of the free radicals are kept under check by the body defense system, the antioxidant system, which includes enzymes and antioxidants that prevent the commencement of oxidative damage and its propagation [8,9]. Ionizing radiation is known to induce oxidative stress through the generation of ROS and FR, thus resulting in the imbalance of the pro-oxidant and antioxidant in the cells, which can break the defense system [10]. The cell structures, including lipids, membranes, proteins and DNA, could be damaged by the overproduction of ROS generated from the interaction between radiation and water molecules in cells [11-14]. Polyunsaturated fatty acids, when exposed to ROS, can be oxidized to hydroperoxides that decompose to hydrocarbons and aldehydes such as malondialdehyde (MDA) [15]. MDA, itself, due to its high cytotoxicity and inhibitory action on protective enzymes, is suggested to act as a tumor promoter and a co-carcinogenic agent [16]. While superoxide dismutase (SOD), the antioxidant enzymes against oxidative damage, catalyzes the dismutation of superoxide anion into H_2_O_2_, which can clear away ROS. Then, H_2_O_2_ can be transformed into H_2_O and O_2_ by catalase. MDA and SOD are two important compounds in charge of the antioxidant system balance.

Now, the bottleneck of radiotherapy is not the lack of cancer-killing means, but the side effect of radiotherapy, which is difficult to overcome. Recently, although there are more and more researches of the protective mechanism of radiation-induced skin injury, the measures of clinical treatment depend mainly on anti-inflammatory, antioxidant [16-18] and cyto-protective, including using hormones, vitamins, Chinese medicine and removal of free radicals. But we are still lack of effective treatment to the radiation-induced skin injury.

*RHIZOMA COPTIDIS* has been reported to exert numbers of pharmacological effect, such as antibacterial [19], and antioxidative [20] and anti-inflammatory [21]. SOD can obviously increase in rats liver treated with *COPTIS CHINENSIS* aqueous extract, which acts as an anti-oxidant agent against CCL4-induced chronic oxidative stress [22]. *COPTIS CHINENSIS* shows strong free radical scavenging activity and stimulate immunity activities [23]. It can increase the expression of antioxidase isozyme induced by the short-time UV-B radiation [24].

The RCE, which is an aqueous extract from *RHIZOMA COPTIDIS* and *COPTIS CHINENSIS*, can ameliorate radiation-induced skin injury in our clinical observation. However, the protective mechanism of RCE in radiation-induced skin injury remains unclear. This study has been initiated to investigate the radioprotective effects of RCE on radiation-induced skin injury in rats.

## Methods

### Plant material and RCE

The *RHIZOMA COPTIDIS* and *COPTIS CHINENSIS* were bought from the traditional Chinese medicine LBX Pharmarcy (Xian, China) and authenticated according to the descriptions found in the Chinese Pharmacopoeia [25] by Dr Sun in the Department of Traditional Chinese Medicine. The voucher samples, ZLK-ZY-07 (*RHIZOMA COPTIDIS*) and ZLK-ZY-13 (*COPTIS CHINENSIS*), were deposited at the Department of Oncology, the Second Affiliated Hospital of Xi’an Jiaotong University.

15 g of *RHIZOMA COPTIDIS* and 15 g of *COPTIS CHINENSIS* were immersed in 800 mL distilled water. After being soaked for 30 min, the *RHIZOMA COPTIDIS* and *COPTIS CHINENSIS* were boiled for 4 h by cease-fire. Then, we used a sterile gauze funnel to lead the liquid extracted into sterile bottles, stored at 4°C for use.

### Animals and treatment

All animals were purchased from Experimental Animal Center of Xi’an Jiaotong University College of Medicine. The animals were housed and handled in strict accordance with the Guidelines of the Institutional and National Committees of Animal Use and Protection. The protocol was approved by the Committee on the Ethics of Animal Experiments of Xi’an Jiaotong University College of Medicine (Certificate No. 22–9601018). All efforts were made to minimize animals’ suffering and to keep the numbers of animals used to a minimum. The animals were randomly divided into 4 groups: control group (C group), treatment group (T group), radiation group (R group), and treatment and radiation group (R + T group). T group and R + T group were treated with 2.5% RCE (0.1ml/cm^2^) twice a day through applichating the area of radiation, 2 d pre-radiation. A single dose of 45 Gy was administered to the right buttock of each rat in R group and R + T group at a dose rate of 1000 cGy/min using a 6MeV-β electronic beam of accelerator (Clinac 2100EX; Varian Medical Systems Inc, CA) at a distance of 100 cm. The irradiated area was 3cm × 3 cm, and the surrounding skin was covered with lead plate. A 1.0 cm tissue equivalent bolus was used to bring the maximal dose to the skin surface. 5 rats of each group were separately executed on 2 d and 49 d post-radiation and then the skin and blood samples were harvested for the assays of malondialdehyde (MDA), superoxide dismutase (SOD) according to the procedures described below. For histopathological study, part of skin was stored in formalin (10%) before use.

### Skin scores

Skin damage was assessed using Semi-quantitative Skin damage scores with scores of 1–5.5 (Table 1) based on previous acute skin damage models [26,27]. Skin damage was measured by unblinded observers approximately daily, and we evaluated it weekly.

**Table 1:** Semi-quantitative Skin damage scores

**SCORE**	**SKIN CHANGES**
1.0	No effect
1.5	Minimal erythema, mild dry skin
2.0	Moderate erythema, dry skin
2.5	Marked erythema, dry desquamation
3.0	Dry desquamation, minimal dry crusting
3.5	Dry desquamation, dry crusting, superficial minimal scabbing
4.0	Patchy moist desquamation, moderate scabbing
4.5	Confluent moist desquamation, ulcers, large deep scabs
5.0	Open wound, full thickness skin loss
5.5	Necrosis

### Histopathological assessment

For the histopathologic study, 5 rats of each group were separately executed on 2 d and 49 d post-radiation and the irradiated fields were obtained from buttock. The skin samples were fixed in 10% formalin. After routine processing, the skin samples were imbedded in paraffin wax. Four-μm-thick slices were prepared and stained with hematoxylin and eosin for evaluation with light microscopy. Damaged areas were evaluated using damage (epidermal atrophy, dermal degeneration such as edema and collagen fiber loss, and hair follicle atrophy) percentages, which were scored on 5-points ordinal scale as follows: Grade 0 = normal, Grade 1 = minimal, Grade 2 = mild, Grade 3 = moderate, Grade 4 = marked, and Grade 5 = severe. The methods were referred to previous studies [28,29].

### Determination of lipid per oxidation

Levels of MDA were determined as described by Yagi [30] with the colorimetric absorption of the thiobarbituric acid-malondialdehyde chromophore used to determine the index of lipid peroxidation.

### SOD activity measurement

Activity of SOD was measured using a commercially available kit purchased from Nanjing Jiancheng BioEngineering (Nanjing, China). SOD activity measurement was based on the instructions of the colorimetric method.

### Statistical analysis

All values were expressed as the mean ± standard deviations (SD). Statistical analysis was performed with student’s *t*-test using the statistical software SPSS 13.0. P < 0.05 was considered as statistically significant.

## Results

### Skin scores

**Figure 1:**
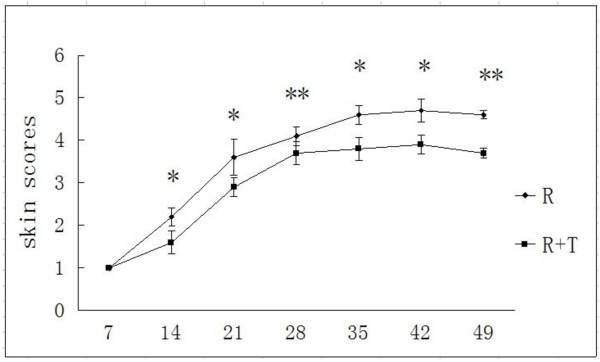
**Skin scores in different time-points. The time courses of the skin scores after 45Gy of irradiation.** All dates are presented as mean ± SD in 5 rats. *P < 0.05, **P < 0.01.

The finding of minimal erythema began in 1 of 5 rats in radiation group (R Group) in week 1 after irradiation. All rats which had received irradiation showed the appearance of erythema together with desquamation on week 2. In R group, skin breakdown could be found in week 3, with dry crusting and minimal scabbing. Patchy moist desquamation emerged in R group in week 4, and then became confluent and enlarged few days later. R group had ulcers and large deep scabs in week 5, and continued to develop. In radiation and treatment group (R + T group), rats showed patchy moist desquamation in week 4 and ulcers in week 6, without any progress later. The rats without irradiation showed no observed remarkable changes in the right buttock skin. Figure 1 showed the skin scores of R group and R + T group at different stages.

### Histological hematoxylin and eosin (HE) staining results

**Figure 2:**
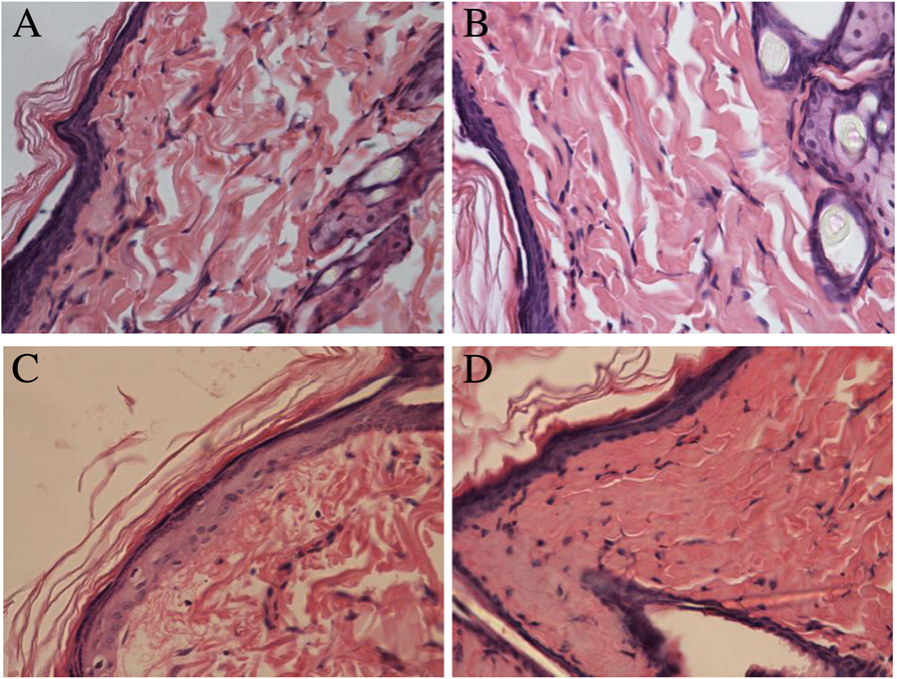
**The histopathological images 2 days after irradiation in the present study. A** and **B**: Normal histopathological images of the skin in the C group and T group. Epidermal, dermal structures and hair follicles were intact; **C**: Radiation injury of the skin in the R group. Epidermal cells and hair follicle epithelial cells swelling, nucleus pycnosis, collagen fiber edema, lead to arrangement disorder, together with congestive vascular reactivity and inflammatory cell infiltration could be found. **D**: Histopathological images of R + T group, reflect a radioprotection against radiation-induced skin damage in terms of epidermal cells swelling, especially in the spine layer and base layer, hair follicle epithelial cells swelling, collagen fiber edema, and nucleus pycnosis. (HE × 400).

2 days after irradiation, insignificant differences of histopathological images were found between C group and T group as shown in Figure 2. Compared with those in C group, all the rats receiving irradiation showed significantly difference in epidermal cells swelling, especially in the spine layer and base layer, hair follicle epithelial cells swelling, collagen fiber edema, which led to arrangement disorder, and nucleus pycnosis. In R group, all those changes were great, compared with those in R + T group, together with visible congestive vascular reactivity in dermis and subcutaneous tissue, inflammatory cell infiltration. The order of tissue damages in each group caused by irradiation was, as follows: R group (3.6 ± 0.55) > R + T group (2.0 ± 0.71) > T group = C group (0).

**Figure 3:**
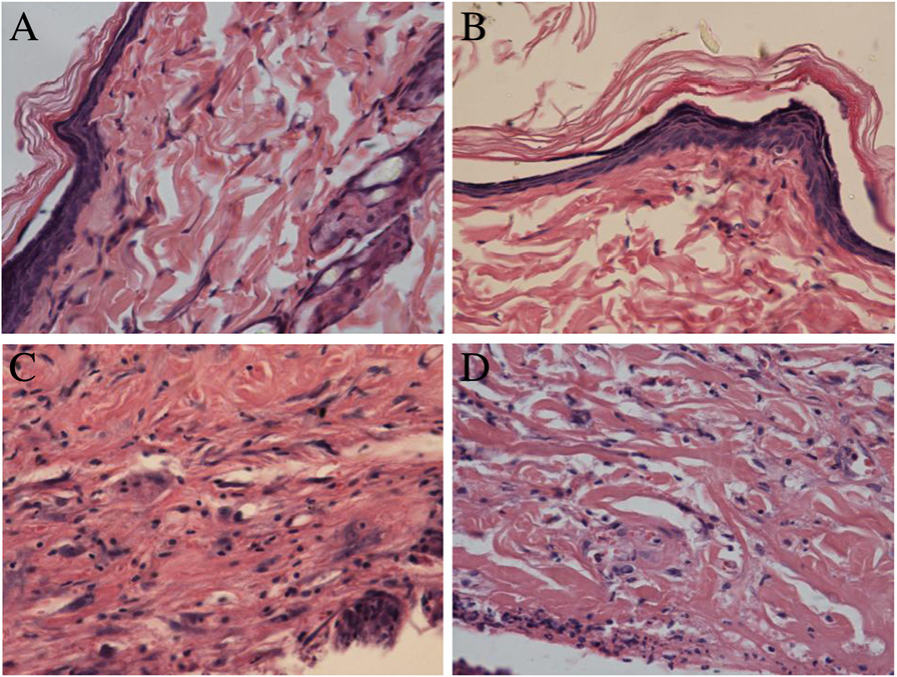
**The histopathological images 49 days after irradiation. A** and **B**: Normal histopathological images of the skin in the C group and T group; **C**: Radiation damage of the skin in the R group. discontinuous dermal structural, with lots of nuclear pyknosis rupture, small amount of new capillaries, collagen fiber dissolution, disordered, inflammatory cells infiltration and few fibroblasts could be found. **D**: Histopathological images of R + T group, reflect a radioprotection against radiation-induced skin damage in terms of continuous dermal structural, lots of new capillaries, more fibroblasts, the lighter collagen fibers change and little amount of inflammatory cells. (HE × 400).

No significant differences were found in the histopathological images between C group and T group 49 d after irradiation. In R group rats, discontinuous dermal structures, with lots of nuclear pyknosis rupture, small amount of new capillaries, collagen fiber dissolution, disordered, inflammatory cells infiltration and few fibroblasts could be found. While in R + T group, continuous dermal structures, lots of new capillaries, more fibroblasts could be observed, together with the lighter collagen fibers change and small numbers of inflammatory cells. The order of tissue damages caused by irradiation was as follows: R group (4.8 ± 0.45) > R + T group (3.2 ± 0.45) > T group = C group (0). Those changes are shown in Figure 3.

### Effects of RCE on Radiation-Induced MDA content in serum and skin

**Figure 4:**
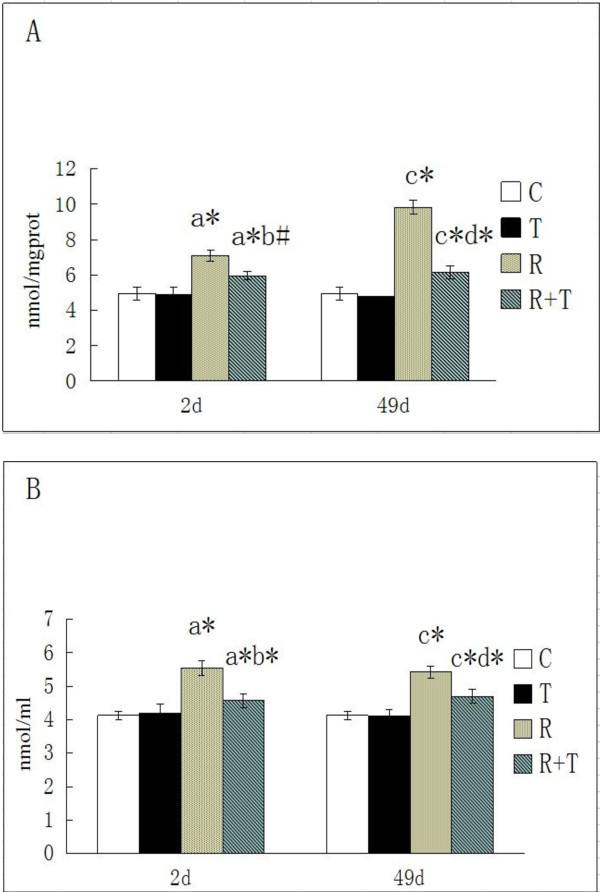
**Effect of RCE on Radiation-Induced MDA in skin (A) and serum (B). a**: compared to C group rats in 2d, **b**: compared to R group rats in 2**d**, **c**: compared to C group rats in 49d, **d**: compared to R group rats in 49d; *p < 0.01, #p < 0.05. None of the skin and serum values of the C and T rats show different significantly (p > 0.05). The cases that significantly differences were found are given in the Figure.

The contents of MDA in serum and skin were 4.13 ± 0.12mmol/l and 4.95 ± 0.35mmol/mgprot in C group. No valuable changes were discovered in T group. The rats receiving radiation showed an increased content of MDA in skin and serum, inhibited by RCE (p < 0.05, compared with that of R group). All data were given in Figure 4. It manifests that RCE may be useful for reducing the generation of MDA.

### Effects of RCE on the induction of radiation-induced antioxidant enzymes in serum and skin

**Figure 5:**
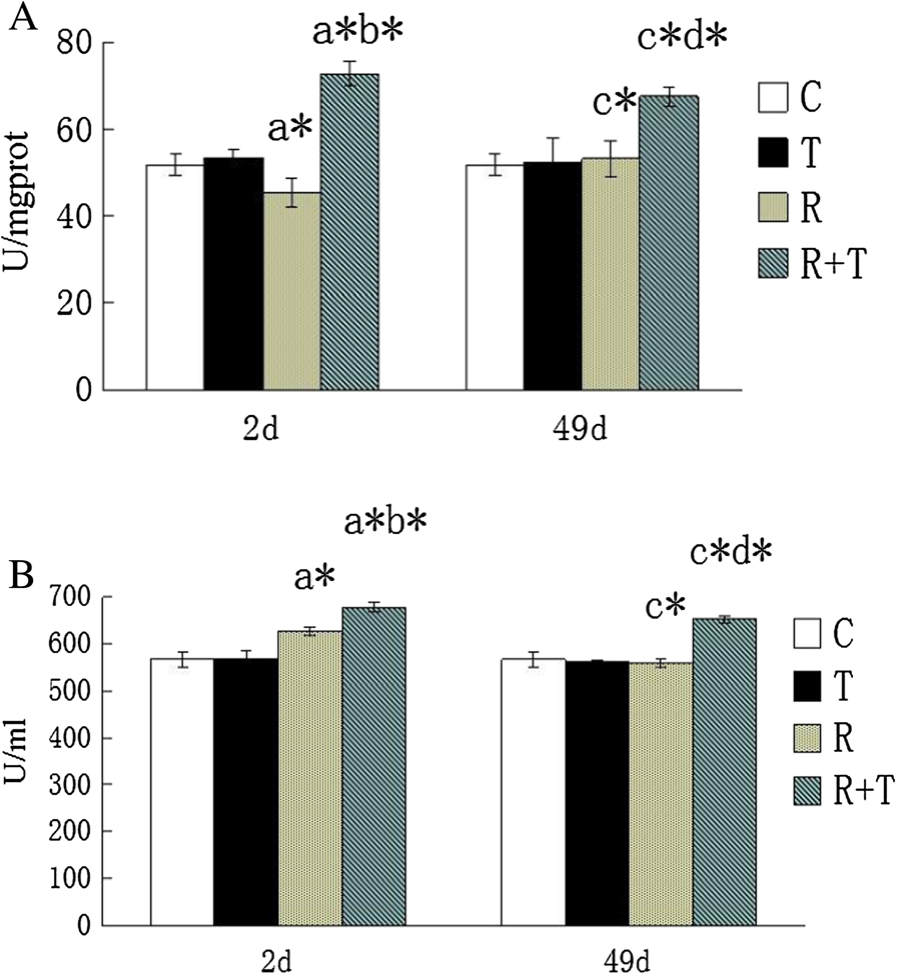
**Effect of RCE on Radiation-Induced SOD in skin (A) and serum (B). a**: compared to C group rats in 2**d**, **b**: compared to R group rats in 2**d**, **c**: compared to C group rats in 49d, **d**: compared to R group rats in 49d; * p < 0.01. None of the skin and serum values of the C and T rats show different significantly (p > 0.05).

The SOD activity of C group in serum and skin were 566.16 ± 15.11U/l and 51.78 ± 2.43U/mgprot as shown in Figure 5. No valuable changes could be found between C group and T group. The skin SOD activity significantly reduced in rats receiving irradiation, compared with rats without irradiation. RCE could inhibit those changes (p < 0.05 compared with that of R group). It indicates that RCE may be useful for scavenging oxygen-derived free radicals.

## Discussion

In the present study, based on the observation of rats receiving a signal dose of 45 Gy of β-ray irradiation, acute skin reactions caused by ionizing radiation started developing on the 7th day post-irradiation in R group. RCE could ameliorate radiation-induced skin injury at different time-points, both in the skin scores and the appear time. As time went by, the protective effect became more predominant. Although RCE could decrease the square of skin injury, no significant difference was found between R group and R + T group. The square of skin injury might be affected by the small numbers of rats, the exact area of radiation and the method of treatment. Through observation and HE staining, we found that the depth of skin injury in R group was deeper than R + T group. The radioprotective effect of RCE might be more related with the depth rather than with the square of skin injury, which needs further study.

HE staining showed the skin damage after irradiation, which was consistent with previous reports [28,31], while it was visible ameliorated after treated with RCE at different time-points. 49 d after irradiation, the rats in R + T group showed more new capillaries and fibroblasts, which played an important role in the skin repair. We believe that RCE can ameliorate radiation-induced skin injury obviously.

MDA and SOD are two important compounds in charge of the antioxidant system balance. The increased MDA and decreased SOD levels can be found in the skin injury caused by irradiation [16]. At the same time-point in the irradiated animals, the marked radiation-induced decrease in SOD activity and rise in MDA content of skin were found in comparison with the controls, while the RCE could ameliorate those changes. A meaningful statistical analysis of SOD values were performed between groups 2 d and 49 d after irradiation, but no statistically significance was found in MDA content. ROS was generated from the interaction between radiation and water molecules, which could cause tissue injury. MDA could be produced from polyunsaturated fatty acids, when exposed to ROS [12]. RCE could ameliorate those changes. As time went by, SOD ceaselessly scavenged FR and ROS. MDA content and SOD activity did not change greatly, and tended to keep dynamic equilibrium in the regulation of internal environment. RCE might cause the phenomenon of no statistical significance in MDA at different time points through the increased SOD content, which could inhibit the production of MDA.

In serum, the increased content of MDA and the decreased content of SOD at different time points recorded in the present study are in agreement with those recorded by Sevil Kilciksiz [12], El-Missiry MA [16], which are inhibited by RCE. The skin exposed to radiation produces large amount of FR and ROS with the chain reaction, and the exchanges of oxidative stress can cause other tissue damage through circulatory system [12,16].

In addition, no marked changes of SOD and MDA, together with the observation and HE staining, were found between C group and T group (Figure 4 and Figure 5) either in skin or in serum. Therefore, RCE is unharmful to rats and maybe a useful drug for radiation-induced skin injury.

## Conclusion

The present study provides evidences for the radioprotective role of RCE against radiation-induced skin damage in rats by modulating oxidative stress in skin, which may be a useful therapy for radiation-induced skin injury.

## Competing interests

The authors declare that they have no competing interests.

## Authors’ contributions

WXJ, LS and WBF designed the research. BMH, MXL contributed to the animal experiments. LS, MXB and LMJ performed the biochemical and Histopathological experiments. KHF and LXX contributed to the reagents, and participated in its design and coordination. LS and DZJ analyzed the data; LS and WBF wrote the paper. Co-first authors: WXJ and LS. All authors have read and approved the final manuscript.

## Pre-publication history

The pre-publication history for this paper can be accessed here:

http://www.biomedcentral.com/1472-6882/13/105/prepub

## References

[B1] Huber R Braselmann H Geinitz H Jaehnert I Baumgartner A Thamm R Figel M Molls M Zitzelsberger H Chromosomal radiosensitivity and acute radiation side effects after radiotherapy in tumour patients–a follow-up study Radiat Oncol 2011 6 32 10.1186/1748-717X-6-32 21473753 PMC3080817

[B2] Sprung CN Chao M Leong T McKay MJ Chromosomal radiosensitivity in two cell lineages derived from clinically radiosensitive cancer patients Clin Cancer Res 2005 11 6352 6358 10.1158/1078-0432.CCR-04-1931 16144940

[B3] Bernstein EF Sullivan FJ Mitchell JB Salomon GD Glatstein E Biology of chronic radiation effect on tissues and wound healing Clin Plast Surg 1993 20 435 453 8324983

[B4] Kouvaris J Kouloulias V Kokakis J Matsopoulos G Myrsini B Vlahos L The cytoprotective effect of amifostine in acute radiation dermatitis: a retrospective analysis Eur J Dermatol 2002 12 458 462 12370135

[B5] Azab KS Mostafa AH Ali EM Abdel-Aziz MA Cinnamon extract ameliorates ionizing radiation-induced cellular injury in rats Ecotoxicol Environ Safety 2011 74 2324 2329 10.1016/j.ecoenv.2011.06.016 21782243

[B6] Simonen P Hamilton C Ferguson S Ostwald P O’Brien M O’Brien P Back M Denham J Do inflammatory processes contribute to radiation induced erythema observed in the skin of humans? Radiother Oncol 1998 46 73 82 10.1016/S0167-8140(97)00115-1 9488130

[B7] Hymes SR Strom EA Fife C Radiation dermatitis: clinical presentation, pathophysiology, and treatment 2006 J Am Acad Dermatol 2006 54 28 46 10.1016/j.jaad.2005.08.054 16384753

[B8] Mansour HH Protective role of carnitine ester against radiation-induced oxidative stress in rats Pharmacol Res 2006 54 165 171 10.1016/j.phrs.2006.04.003 16757176

[B9] Juránek I Bezek S Controversy of free radical hypothesis: reactive oxygen species–cause or consequence of tissue injury? Gen Physiol Biophys 2005 24 263 278 16308423

[B10] Srinivasan M Sudheer AR Pillai KR Kumar PR Sudhakaran PR Menon VP Modulatory effects of curcumin on γ-radiation-induced cellular damage in primary culture of isolated rat hepatocytes Environ Toxicol Pharmacol 2007 24 98 105 10.1016/j.etap.2007.03.001 21783796

[B11] Watters D Molecular mechanisms of ionizing radiation-induced apoptosis Immunol Cell Biol 1999 77 263 271 10.1046/j.1440-1711.1999.00824.x 10361259

[B12] Kilciksiz S Demirel C Erdal N Gürgül S Tamer L Ayaz L Ors Y The effect of N-acetylcysteine on biomarkers for radiation-induced oxidative damage in a rat model Acta Med Okayama 2008 62 403 409 19122686 10.18926/AMO/30946

[B13] Valko M Leibfritz D Moncol J Cronin MT Mazur M Telser J Free radicals and antioxidants in normal physiological functions and human disease Int J Biochem Cell Biol 2007 39 44 84 10.1016/j.biocel.2006.07.001 16978905

[B14] Flora SJ Role of free radicals and antioxidants in health and disease Cell Mol Biol (Noisy-le-grand) 2007 53 1 2 17535753

[B15] Mathews WR Guido DM Fisher MA Jaeschke H Lipid peroxidation as molecular mechanism of liver cell injury during reperfusion after ischemia Free Radic Biol Med 1994 16 763 770 10.1016/0891-5849(94)90191-0 8070679

[B16] El-Missiry MA Fayed TA El-Sawy MR El-Sayed AA Ameliorative effect of melatonin against gamma-irradiation-induced oxidative stress and tissue injury Ecotoxicol Environ Safety 2007 66 278 286 10.1016/j.ecoenv.2006.03.008 16793135

[B17] Sinha M Das DK Bhattacharjee S Majumdar S Dey S Leaf Extract of Moringa oleifera Prevents Ionizing Radiation-Induced Oxidative Stress in Mice J Med Food 2011 14 1167 1172 10.1089/jmf.2010.1506 21861723

[B18] Liu Y Zhang H Zhang L Zhou Q Wang X Long J Dong T Zhao W Antioxidant N-acetylcysteine attenuates the acute liver injury caused by X-ray in mice Eur J Pharmacol 2007 575 142 148 10.1016/j.ejphar.2007.07.026 17825281

[B19] Kong WJ Zhao YL Xiao XH Wang JB Li HB Li ZL Jin C Liu Y Spectrum-effect relationships between ultra performance liquid chromatography fingerprints and anti-bacterial activities of Rhizoma coptidis Anal Chim Acta 2009 634 279 285 10.1016/j.aca.2009.01.005 19185133

[B20] Jung HA Min BS Yokozawa T Lee JH Kim YS Choi JS Anti-Alzheimer and antioxidant activities of Coptidis Rhizoma alkaloids Biol Pharm Bull 2009 32 1433 1438 10.1248/bpb.32.1433 19652386

[B21] Kim JM Jung HA Choi JS Lee NG Identification of anti-inflammatory target genes of Rhizoma coptidis extract in lipopolysaccharide-stimulated RAW264.7 murine macrophage-like cells J Ethnopharmacol 2010 130 354 362 10.1016/j.jep.2010.05.022 20546869

[B22] Feng Y Wang N Ye X Li H Feng Y Cheung F Nagamatsu T Hepatoprotective effect and its possible mechanism of Coptidis rhizoma aqueous extract on carbon tetrachloride-induced chronic liver hepatotoxicity in rats J Ethnopharmacol 2011 138 683 690 10.1016/j.jep.2011.09.032 21963555

[B23] Yan H Sun X Sun S Wang S Zhang J Wang R An P Yang F Kang W Anti-ultraviolet radiation effects of Coptis chinensis and Phellodendron amurense glycans by immunomodulating and inhibiting oxidative injury Int J Biol Macromol 2011 48 720 725 10.1016/j.ijbiomac.2011.02.014 21356237

[B24] Wen Q Zhang N Cao RX Zhou XY Tang J Wu NB Response of isozyme and stress indexes of Coptis chinensis to UV-B radiation Zhong Yao Cai 2012 35 341 346 22876667

[B25] Anon Pharmacopoeia of The People’s Republic of China Edited by 2010 Chinese 2010 Beijing: China Medical Science Press 285 287

[B26] Field SB Law MP The relationship between early and late radiation damage in rodents’skin Int J Radiat Biol Relat Stud Phys Chem Med 1976 30 557 564 10.1080/09553007614551411 1087295

[B27] Kumar S Kolozsvary A Kohl R Lu M Brown S Kim JH Radiation-induced skin injury in the animal model of scleroderma: implications for post-radiotherapy fibrosis Radiat Oncol 2008 24 40 19025617 10.1186/1748-717X-3-40 PMC2599892

[B28] Kitagawa J Nasu M Okumura H Shibata A Makino K Terada H Matsumoto S Allopurinol Gel Mitigates Radiation-induced Mucositis and Dermatitis J Radiat Res 2008 49 49 54 10.1269/jrr.07038 18094531

[B29] Abe Y Urano M Fraction size-dependent acute skin reaction of mice after multiple twice-a-day doses Int J Radiat Oncol Biol Phys 1990 18 359 364 10.1016/0360-3016(90)90101-O 2303366

[B30] Yagi K A simple fluorometric assay for lipoperoxide in blood plasma Biochem Med 1976 15 212 216 10.1016/0006-2944(76)90049-1 962904

[B31] Ertekin MV Tekin SB Erdogan F Karslioglu I Gepdiremen A Sezen O Balci E Gündogdu C The effect of zinc sulphate in the prevention of radiation-induced dermatitis J Radiat Res 2004 45 543 548 10.1269/jrr.45.543 15635264

